# Happiness

**Published:** 1871-10

**Authors:** 


HAPPINESS. — To be happy, and to live long, the three grand essentials are: Be busy, love somebody, and have high aims.

